# Supplementary material for: Payoff information hampers the evolution of cooperation
Source: Nat Commun. 2017 May 2;8:15147. doi: 10.1038/ncomms15147 (PMC5418570; doi:10.1038/ncomms15147)
Supplement: Supplementary Information — Supplementary Figures, Supplementary Table, Supplementary Discussion and Supplementary Methods. [file ncomms15147-s1.pdf]

Supplementary Information

Article: Payoff Information Hampers the Evolution of Cooperation

Authors: Steffen Huck, Johannes Leutgeb, Ryan Oprea

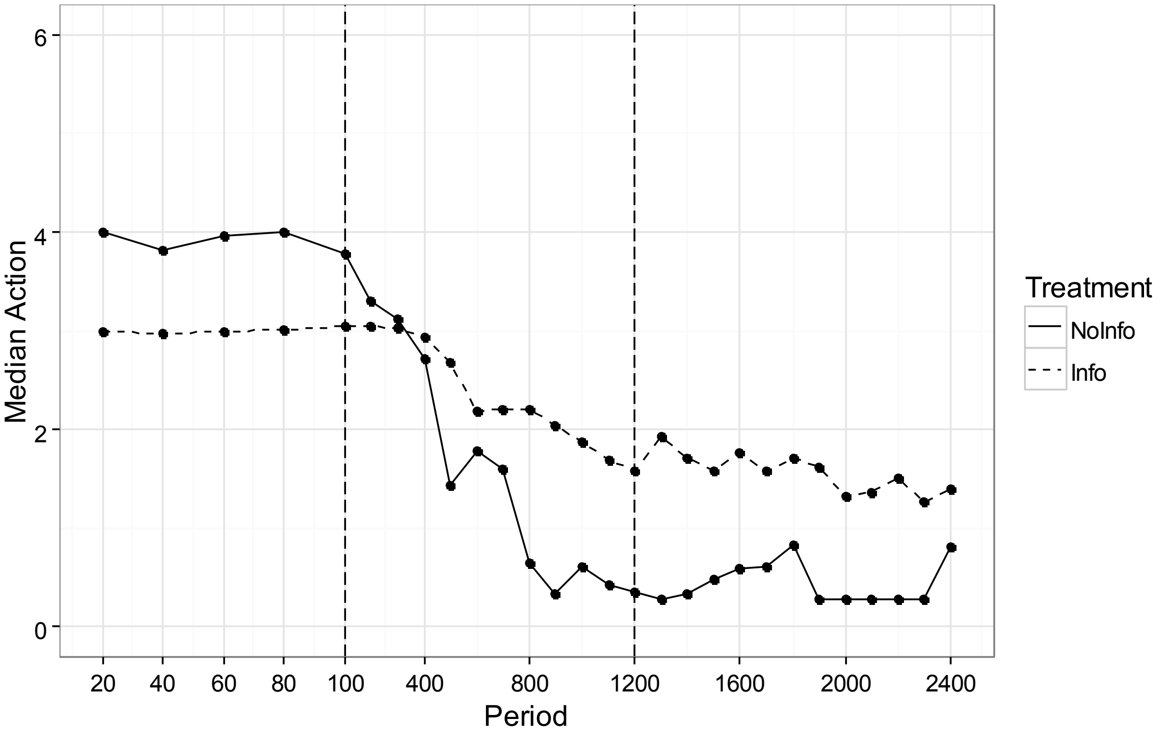

Supplementary Figure 1 Median Actions with 2 seconds per period. For comparability the cut-offs chosen are equivalent in clock time to the cut-offs in the treatment with 8 seconds. Up to period 100 each dot represents the median action in bins of 20 periods. From period 100 (dashed vertical line) on each dot represents the median action in bins of 100 periods. In the naïve phase (periods 1-100) the average action (standard deviation in parentheses) is 3.89 (1.44) in NoInfo and 3.06 (1.16) in Info. In the learning phase (periods 101-1200) this changes to 2.06 (2.04) in NoInfo and 2.42 (1.42) in Info. Finally, in the long-run phase (periods 1200-2400) behaviour settles down at 1.36 (1.71) in NoInfo and 1.75 (1.47) in Info.

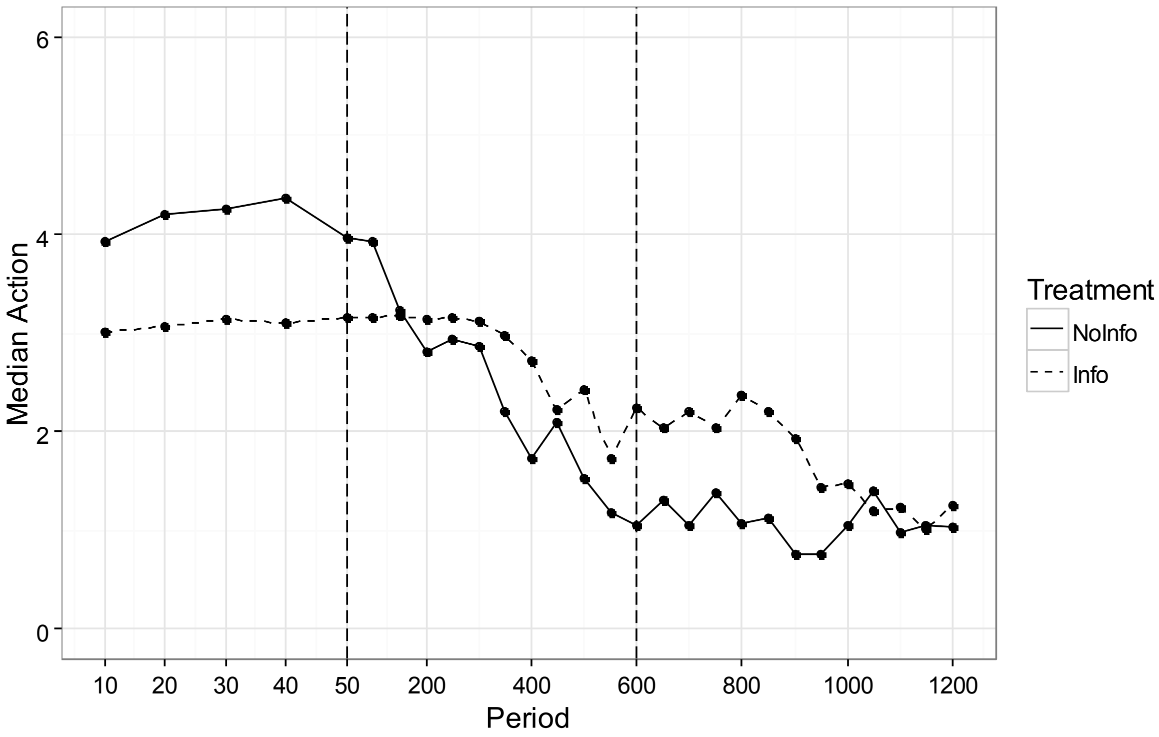

Supplementary Figure 2. Median Actions with 4 seconds per period. For comparability the cut-offs chosen are equivalent in clock time to the cut-offs in the treatment with 8 seconds. Up to period 50 each dot represents the median action in bins of 10 periods. From period 50 (dashed vertical line) on each dot represents the median action in bins of 50 periods. In the naïve phase (periods 1-50) the average action (standard deviation in parentheses) is 3.86 (1.51) in NoInfo and 3.33 (1.18) in Info. In the learning phase (periods 51-600) this changes to 2.54 (1.94) in NoInfo and 2.74 (1.71) in Info. Finally, in the long-run phase (periods 601-1200) behaviour settles down at 1.49 (1.49) in NoInfo and 2.05 (1.75) in Info.

	Dependent variable:		
	CHANGE		
	(1) Periods 1-25	(2) Periods 26-300	(2) Periods 300-600
COPY UP	0.422***	0.325***	0.219**
	(0.144)	(0.094)	(0.084)
COPY DOWN	0.015	-0.135	-0.320***
	(0.172)	(0.105)	(0.113)
WCLR	0.062*	0.116**	0.097
	(0.031)	(0.042)	(0.067)
BR	0.114***	0.099**	0.082
	(0.037)	(0.042)	(0.073)
INFO	0.355*	-0.152	-0.239*
	(0.186)	(0.129)	(0.136)
COPY UP:INFO	-0.518***	0.088	0.161
	(0.168)	(0.122)	(0.114)
COPY DOWN:INFO	-0.362*	0.213	0.365**
	(0.196)	(0.132)	(0.137)
WCLR:INFO	-0.006	-0.061	-0.068
	(0.053)	(0.052)	(0.075)
BR:INFO	0.226***	0.107	0.110
	(0.066)	(0.063)	(0.085)
Constant	0.264	0.278***	0.422***
	(0.161)	(0.099)	(0.121)
Observations	1,428	14,300	11,916
R^2	0.233	0.243	0.260
F Statistic	47.91***	138.03***	35.66***
	*p < 0.1 **p < 0.05 ***p < 0.01		
Note:	Standard errors clustered on groups in parentheses		

Supplementary Table 1 Linear Probability Model Estimates. This table is similar to Table 1 but instead of pooling periods 26-600 it reports separate results for the learning phase (26-300) and the long run phase (periods 300-600). The results remain qualitatively similar.

Supplementary Discussion

In the treatments with 2 and 4 seconds per period similar procedures as in the treatment with 8 seconds per period were applied. Total time spent in the environment was kept constant at 80 minutes but subjects interacted with a higher frequency. The payoff function in a period was the same as before but points were converted to euro by dividing by 4000 and 8000 respectively. Subjects in both treatments exhibit behaviour similar to the behaviour observed in the original treatment with 8 seconds per period. For comparability we choose cut-offs that are equivalent in clock time to the cut-offs in the treatment with 8 seconds, i.e. periods 50 and 600 in the treatment with 4 seconds and periods 100 and 1200 in the treatment with 2 seconds. In the naïve phase subjects start out with significantly higher choices in NoInfo than in Info in both treatments (two-sided MWU tests, $p < 0.001$). In the learning phase again choices start to drop massively. In the long run then subjects in NoInfo choose significantly more cooperate outcomes in both treatments (two-sided MWU tests, $p < 0.1$). Though this difference is significant in both cases, average behaviour is not as close to the one-shot Nash in either case as it is in the Info treatment reported in the main text. This is due to increased heterogeneity (note the high standard deviations reported in the Figure captions): though most groups converge to Nash-like behaviour, a minority of groups in each case manage to move away from Nash into collusive territory.

Supplementary Methods

Instructions

This section contains the instructions that subjects received in the Info treatment. The instructions are translated from German for review purposes. Instructions in the NoInfo treatment are the same except for a different screenshot and minus the sentence mentioning the black line.

Instructions

Welcome! Thank you for participating in this economic experiment. If you read these instructions carefully, you can earn a non-trivial amount of money. The money that you earn during the course of this experiment will be paid to you in cash at the end of the last period. Please remain quiet and do not look at the screens of the other participants. If you have questions or if you need help, please give us a hand sign and we will come to your place. If you disrupt the experiment by speaking, laughing, etcetera, we will exclude you from the experiment without payment. We expect and appreciate your cooperation.

All procedures in the experiment will take place exactly as they are described in these instructions.

Basic structure of the experiment

In this experiment the computer will match you anonymously with another player. The experiment is divided into periods. In each period you and the other player will secretly choose actions. The combination of actions that you and your partner have chosen at the end of the period will determine the amount of points that you earn in this period.

We will not explain to you exactly how your points are calculated, but here are some hints:

Your points in each period are determined solely by your strategy and the strategy of your counterpart.

The function that determines your points will not change during the experiment. If you and your counterpart choose the same actions at some points in time A and B, you will earn the same amount at point A as in point B.

Your payoff function is symmetric to your counterpart's function. If you and your counterpart choose the same action in the same period, you will earn the same amount of points.

Computer Display

Figure 1 shows the display which you will use to make choices and through which you will interact with your counterpart. At the top of the display you see a progress bar that shows how much time has passed in the current period. When the bar is full the period ends and another period starts immediately. Your action is the position (from the left to the right) of the black square at the lower part of the display. During a period you can change your preliminary action freely by moving the square like a slider to the left and to the right, or by clicking on the desired

position. Your actual action for the entire period is only determined by the position of the slider at the end of the period.

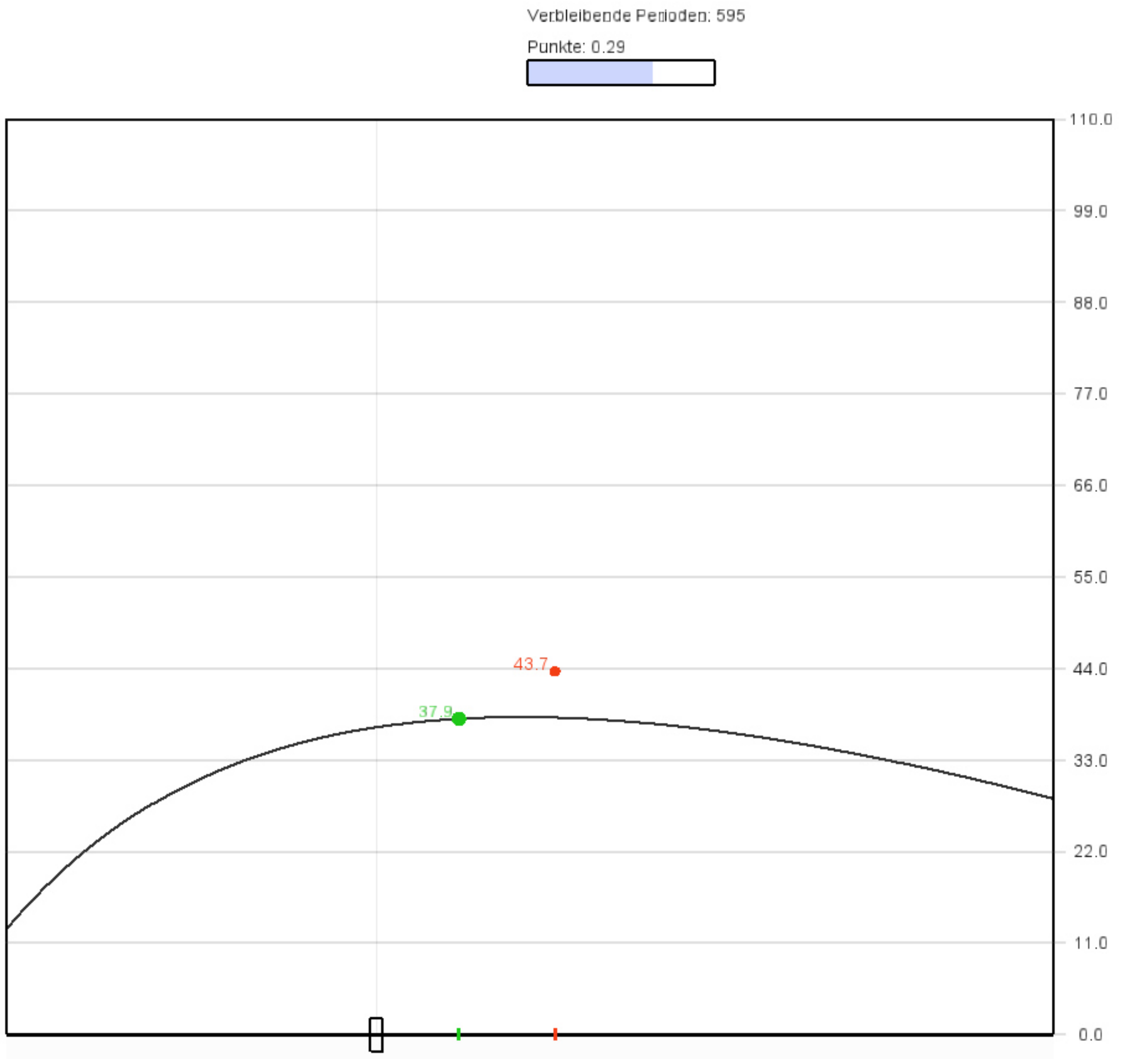

Figure 1: The Interface

After a period has ended you will see a green point that shows the amount of points that you earned in the previous period. The higher the point the more points you have earned. The exact number of points is shown next to the point. At the same time you will see a red mark at the bottom of the display which shows the action of your counterpart in the previous period. You will also see a red point that shows the amount of points that your counterpart earned in the previous period. Next to this point you will also see the number of points that your counterpart earned. Finally you will see a black line that shows you how many points you could have earned at each position of the slider, depending on the action that your counterpart has chosen in the previous period.

It is important for you to understand that the action of your counterpart, your points, and the points of your counterpart are always the results of the previous period. You will not receive any information about the points or the action of your counterpart in the current period, until it has passed.

Earnings

In this experiment you will first earn points that are then converted into Euro at a rate of 0.3 Euro per point and paid out to you in cash. The exchange rate is noted down on the whiteboard at the end of the room. The earnings that are shown to you at the end of the period are the amount of points that you would earn in the entire experiment if you and your counterpart would decide the same in all periods.

Your points will accumulate over the course of the experiment. The points that you have already earned are shown at the upper end of the display.

If you have not understood something, please raise your hand. We will answer your questions personally.

Thank you for your participation!
